# From pre-oral secretions to gut digestion: How do *Lucilia sericata* (Diptera: Calliphoridae) larvae handle *Leishmania major*? 

**DOI:** 10.1371/journal.pone.0334553

**Published:** 2025-10-22

**Authors:** Azam Malekian, Naseh Maleki-Ravasan, Somayeh Mohammadi, Ali Khamesipour, Parviz Parvizi

**Affiliations:** 1 Department of Parasitology, Pasteur Institute of Iran, Tehran, Iran; 2 Center for Research and Training in Skin Diseases and Leprosy, Tehran University of Medical Sciences, Tehran, Iran; Cairo University Faculty of Veterinary Medicine, EGYPT

## Abstract

Zoonotic cutaneous leishmaniasis (ZCL), caused by *Leishmania major*, is a neglected tropical disease affecting impoverished populations. Current treatments are limited by cost, resistance, and side effects, highlighting the need for affordable, sustainable interventions. *Lucilia sericata* larvae, used in maggot therapy, effectively treat chronic wounds through debridement, antimicrobial activity, and healing promotion. This study explores how *L. sericata* processes *L. major* and proposes its potential application in ZCL treatment. The life cycles of *L. sericata* and *L. major* were maintained in laboratory conditions. Larval-parasite interactions were tested across substrates [hen liver, rat spleen, Roswell Park Memorial Institute (RPMI) 1640 cell culture medium] and time intervals (30–240 minutes). Extracorporeal effects were evaluated using trypan blue exclusion and MTT (3-(4,5-dimethylthiazol-2-yl)-2,5-diphenyltetrazolium bromide) assays; intracorporeal interactions via microscopy and nested-PCR targeting *L. major* rRNA genes. *L. sericata* excretion/secretion products and microbiota exhibited strong anti-leishmanial activity. Promastigotes were deformed within 1 hour post-exposure (hpe), fully inactivated at 4 hpe, and lysed by 6 hpe. In RPMI medium, the treatment group (*L. sericata* + *L. major*) showed significant reductions in active parasites and viable cells compared to controls after 4 hours. Microscopy revealed no parasites in larval guts, but PCR detected *L. major* DNA in all specimens, suggesting partial digestion. This study demonstrates that *L. sericata* can eliminate *L. major* through intra- and extra-oral digestion, supporting its potential as a biotherapeutic agent for ZCL-associated wounds. These findings offer a foundation for developing larval therapy protocols in dermatology. Further studies in animal models and clinical trials are required to validate this approach for managing ZCL.

## Introduction

The green bottle fly *Lucilia sericata* (Diptera: Calliphoridae), a common causative agent of facultative myiasis, has long been recognized for its crucial role in maggot debridement therapy (MDT). This biosurgical approach is applied to remove necrotic tissues, disinfect wounds, and promote tissue regeneration [1,2]. The key aspect of this process is the larva’s ability to secrete digestive enzymes and antimicrobial compounds while expelling gut microbiota onto substrates before ingestion, followed by vigorous enzymatic digestion within the gut [3–5]. These mechanisms facilitate the breakdown of necrotic tissue and contribute to the suppression or elimination of infectious agents in the wound environment [2]. Among the wound pathogens, kinetoplastid protozoan microorganisms such as *Leishmania major*—the causal agent of ZCL—present a significant challenge due to their complex life cycle, immune evasion strategies, environmental resilience, and the socio-economic barriers associated with their control [6–10].

As a neglected tropical disease, ZCL, remains a major public health concern in endemic areas, affecting approximately 350 million people and resulting in 2 million new cases annually [11]. The transmission cycle of the parasite involves sandflies as vectors and mammals as hosts, often giving rise to chronic skin lesions that are difficult to treat [12,13]. While current therapeutic options are available, they are often limited by high costs, drug resistance, and adverse side effects [14]. Consequently, alternative strategies for managing *Leishmania* infections, particularly those that utilize natural biological systems, have garnered increasing attention [12,15].

*Lucilia sericata* larvae could offer a promising solution to these challenges. Their pre-oral secretions, which are rich in proteolytic enzymes and antimicrobial peptides [3,16,17], can degrade the extracellular components of *L. major* promastigotes or disrupt their surface structures. The larval gut environment, characterized by acidic pH, digestive enzymes, and interactions with gut microbiota [5,18,19], creates an inhospitable environment for ingested parasites. Experiments involving various strains of the Old and New World *Leishmania* parasites—extracellular promastigote and intracellular amastigote forms—along with in vivo and in vitro data, animal and human models, and larval excretion/secretion (ES) products and whole-body products, have demonstrated the potential of *L. sericata* larvae to inhibit the activity and viability of *L. major* and promote healing in difficult-to-treat ZCL wounds [20–27].

Despite the growing attention to the therapeutic applications of MDT, little is known about the mechanisms through which *L. sericata* larvae interact with protozoan parasites such as *L. major*. This knowledge gap underscores the need for comprehensive studies that investigate both the pre-oral and oral digestion phases in parasite processing. By elucidating how *L. sericata* larvae degrade or inactivate *L. major*, we can gain valuable insights into novel strategies for managing neglected tropical diseases such as ZCL. The current study aimed to explore the complex interplay between larval ES products, gut physiology, and parasite survival, shedding light on the broader implications of MDT in infectious disease management. Hence, a preliminary protocol for larval therapy is recommended for the effective management of ZCL.

## Materials and methods

### Parasite culture

Promastigote forms of *L. major* (MRHO/IR/75/ER) were obtained from the Department of Parasitology, Pasteur Institute of Iran. The *Leishmania* culture media were prepared according to the instructions described in the literature [28,29]. Parasites were initially cultured in the supportive Novy-MacNeal-Nicolle medium and then supplemented with complete RPMI 1640 with 10% fetal bovine serum after centrifugation. After every 10 consecutive subcultures in RPMI, we injected 1 × 10^6^ stationary phase *L. major* promastigotes into BALB/c mice to maintain their infectivity. The obtained parasites were kept at 25°C for further use.

### Mass rearing and preparation of *L. sericata*

A colony of *L. sericata* (Tehran strain) was maintained in the insectary conditions within cages (45 × 45 × 45 cm) under controlled conditions at 25 ± 5°C, 16:8 h light/dark photoperiod, and 45 ± 5% relative humidity. Adult flies were fed cotton balls saturated with 20% sugar (sucrose and honey 50 w/w) solution and date paste. Larvae were grown in substrates consisting of chicken liver, and before pupation, they were transferred to a pot containing sawdust. Representative fly specimens were occasionally selected from the colonies and checked for identity using standard identification keys [30]. The second instar larvae were selected for exposure to the *Leishmania* parasite due to their high nutritional activity and efficiency in MDT [31]. Before the interaction, to surface sterilize, the second instar larvae specimens were immersed and shacken consecutively in cold prepared, distilled water, 5% sodium hypochlorite, 70% ethanol for 3 min. They were finally rinsed vigorously with distilled water.

### Interaction of larvae with parasite in a controlled setting

**Fig 1:**
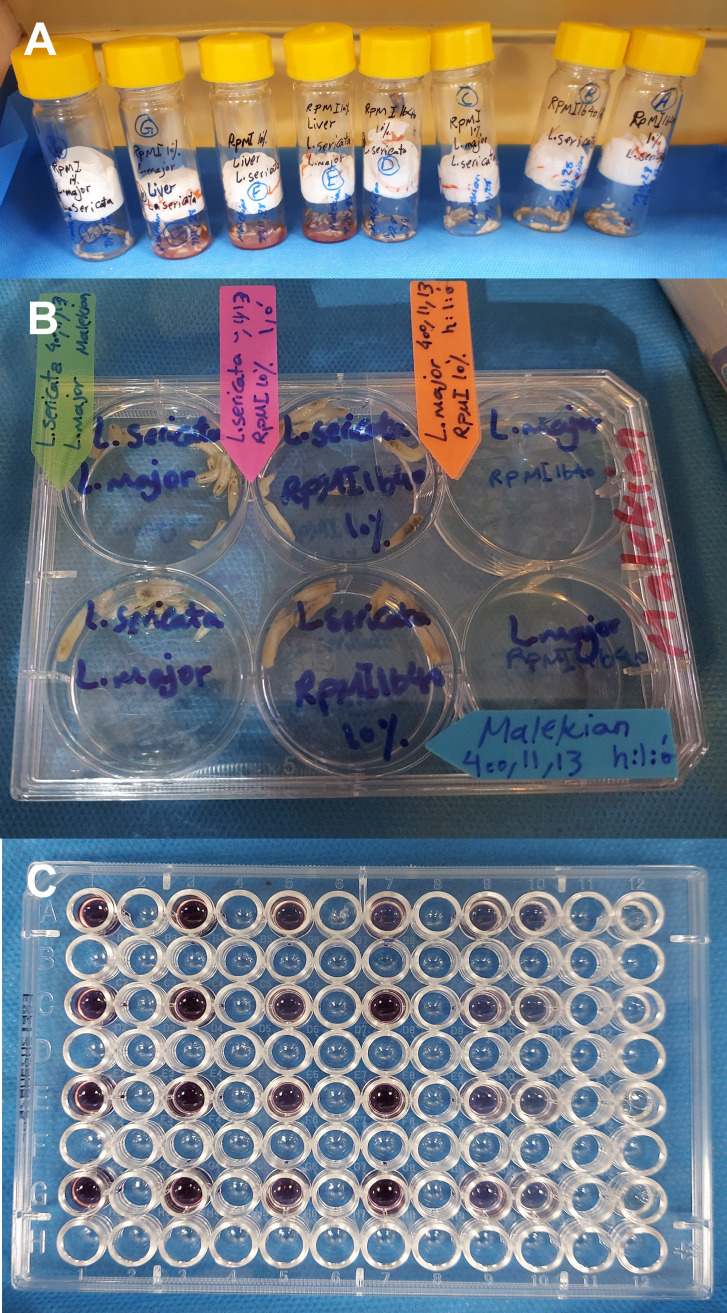
Steps for designing and conducting the larvae-parasites interaction experiment. A) Confining the larval range of motion within a restricted area using physical barriers to increase the likelihood of encountering the parasite; B) Direct interaction between larvae and parasites in a controlled environment for a specified time period (24 hours) under standard laboratory conditions; C) Determining the number of viable cells in study groups using the MTT assay, where purple color indicates formazan crystals produced by metabolically active viable cells.

The reaction of *L. sericata* larvae to *Leishmania* parasites, whether through direct interaction or ingestion, has not been investigated in vitro. To test these hypotheses, we conducted a cross-sectional study. First, a suitable substrate for the growth of both larvae (chicken liver, and rat spleen) and parasites (RPMI and rat spleen) was selected. A volume of 150 μl of RPMI containing 24 × 10^6^ stationary phase promastigotes was added to each medium containing 5 larvae, 5 g of animal tissue, or 1 ml of RPMI). To ensure that the larvae were exposed to the *Leishmania* parasites, we limited the space between the glass filter top tubes using a net and sterile sponge (Fig 1A). Following the larva-parasite interaction was initiated, 20 μl of the medium was sampled every 30 minutes. Afterward, the activity and viability of the parasites were checked under a microscope and 40 × magnification using a hemocytometer to determine the optimal duration for the interaction. The exposure environment in which both larvae and parasites survived until the end of the interaction was identified as the appropriate environment for further studies. The assay included four replicates for each medium.

### Larval-parasite extracorporeal interactions

After selecting the appropriate time and medium, three interacting groups were determined as follows: group 1 consisted of a medium containing five larvae and 24 × 10^6^ *L. major* parasites, group 2 comprised a medium containing 24 × 10^6^ *L. major* parasites, and group 3 included a medium containing five larvae (Fig 1B). After 4 hours of exposure (the maximum time the parasite was active in the selected medium) at room temperature, 20 μl of the samples were examined under a microscope. The viability and activity of parasites were determined using trypan blue exclusion and MTT [3-(4,5-dimethylthiazol-2-yl)-2,5-diphenyltetrazolium bromide] assays, respectively (Fig 1C). To evaluate the viability of the parasites, we mixed 50 μl of larval-parasite interaction medium with 50 μl of trypan blue 0.4%, and after 10 minutes, 20 μl of the mixture was examined on a hemocytometer. Parasites that turned blue were classified as non-viable (dead) cells. An MTT assay was performed to assess cell activity and differentiate between healthy and unhealthy cells. To this end, 100 μl of interaction medium was added to each well of a 96-well plate. Subsequently, 10 μl of prepared MTT was introduced to the wells, and the plate was incubated at 24°C for 4 hours. The plate was centrifuged at 2000 RCF at room temperature for 5 minutes. The supernatant of the wells was replaced with 100 μl of DMSO. After 5 minutes, the absorbance of the samples was recorded using an ELISA Reader (Organon Teknika, Netherlands) at a wavelength of 496 and 600 nm. The assay included three replicates for each group.

### Larval-parasite intracorporeal interactions

To determine whether the larvae feed on *Leishmania* parasites, we assessed the presence of these parasites within their bodies using both microscopic and molecular methods. The larvae were first surface-sterilized by washing them with 70% ethanol, followed by rinsing with phosphate-buffered saline for three minutes. Subsequently, their digestive tracts were dissected on a glass slide under a microscope after immobilizing the larvae by placing them on ice. The intestines were then gently disrupted using a syringe needle. The resulting preparations were fixed and stained with Giemsa stain. Finally, the presence or absence of parasites was examined under a microscope using a 40 × objective lens. Total DNA was extracted from individual gut samples using a universal kit (Cat No. DX0015, AnaCell, Iran), following the manufacturer’s protocol. *Leishmania* ribosomal DNA and 5.8S sequences available in the GenBank were utilized to design two pairs of primers: 5′-AAGCTCTATTGTGTCATCCCC-3′ (ExF) and 5′-CGCGTGTGTATTTGTCCAAC-3′ (ExR) and 5′-TGCCATATTCTCAGTGTCGAAC-3′ (InF) and 5′-CTGACTTGTCTACGTGTGC-3′ (InR). These primers were employed to amplify regions of 213 bp and 166 bp of the gene, respectively. The PCR reaction was conducted in a 50 µL reaction volume containing 50 ng of DNA, 10 pmols of each primer, 1 mM of dNTPs, 1 U of Taq DNA polymerase (CinnaGen Company, Tehran, Iran), and a PCR buffer. The PCR conditions consisted of an initial denaturation at 95°C for 5 minutes, followed by 40 cycles of 95°C for 30 seconds, 60°C for 30 seconds, and 72°C for 30 seconds, with a final extension step at 72°C for 10 minutes. The resulting amplicons were separated by 1.5% (w/v) agarose gel electrophoresis in TAE buffer, stained with DNA Safe Stain, and visualized using a UV transilluminator.

### Statistical analysis

Statistical analysis of the results were performed using GraphPad Prism 10.3.0.507 software. The analyses included one-way ANOVA, Mixed Model ANOVA, and t-tests. In all experiments, differences with a *p* value less than 0.05 (*p* < 0.05) were considered statistically significant.

## Results

### Time and appropriate substrate for larvae and parasite exposure

**Fig 2:**
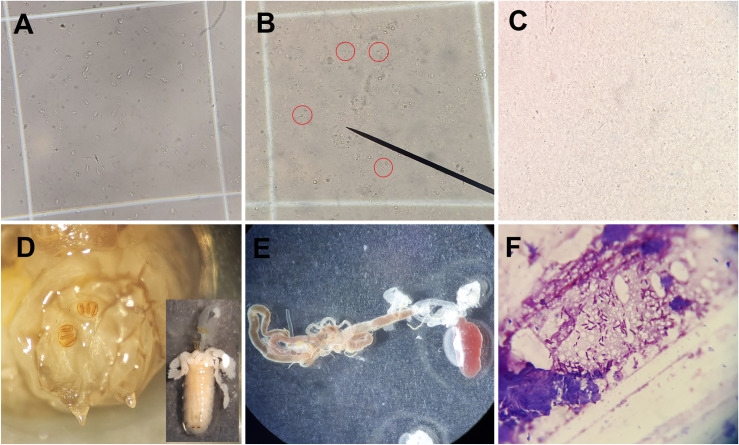
Pre-oral (A-C) and oral (D-E) digestion of *Leishmania major* by *Lucilia sericata* larvae. A: Deformation of *L. major* induced by larval excretion/secretion products; B: lysis of deformed parasites; C: comprehensive lysis of parasites within the interactive environment; D: micro-dissection of larvae exposed to the parasite (middle: third instar spiracles; right: salivary glands connected to the digestive canal); E: complete alimentary canal; F: gut tissues of the larvae stained with Giemsa, showing the presence of some bacteria but no visible *L. major*.

**Fig 3:**
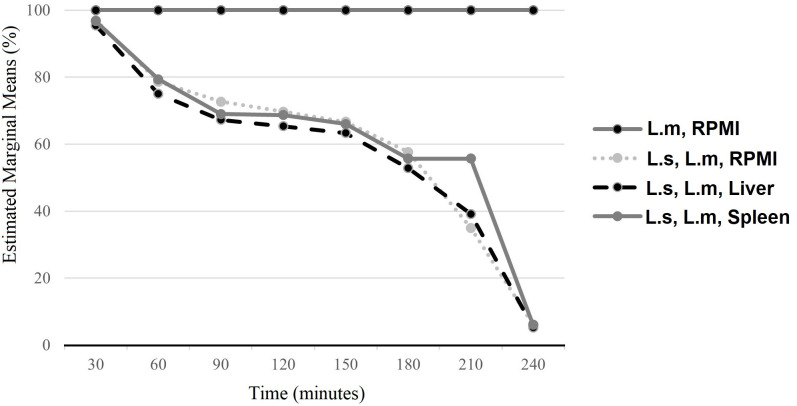
Comparison of the average survival percentages of parasites across different study groups at consecutive 30-minute intervals to determine the optimal duration for larval-parasite interactions. A one-way analysis of variance (ANOVA) test, corrected using a Mixed Model ANOVA, was applied at a 95% confidence level to assess variations among the groups (Larvae/Parasites/Liver, Larvae/Parasites/Spleen, and Larvae/Parasites/RPMI).

Microscopic observations of various treatments (larvae/parasite/liver, larvae/parasite/spleen, larvae/parasite/RPMI) revealed that one hour after the interaction, the parasites began to deform—characterized by wrinkling, shortening of the parasite body, and retraction of the flagellum. The parasites became inactive after four hours and were completely lysed by six hours (Fig 2A-C). A Mixed Model ANOVA was employed to compare the average survival percentages of the study groups at consecutive 30-minute intervals. Throughout the study, the survival percentage of parasites in the control group remained consistent, showing no changes over time. In contrast, the intervention groups demonstrated a significant reduction in survival percentages across time intervals from 30 to 240 minutes (*p* < 0.001; Fig 3). Based on these findings, the interaction time between larvae and parasites was determined to be four hours of exposure.

**Fig 4:**
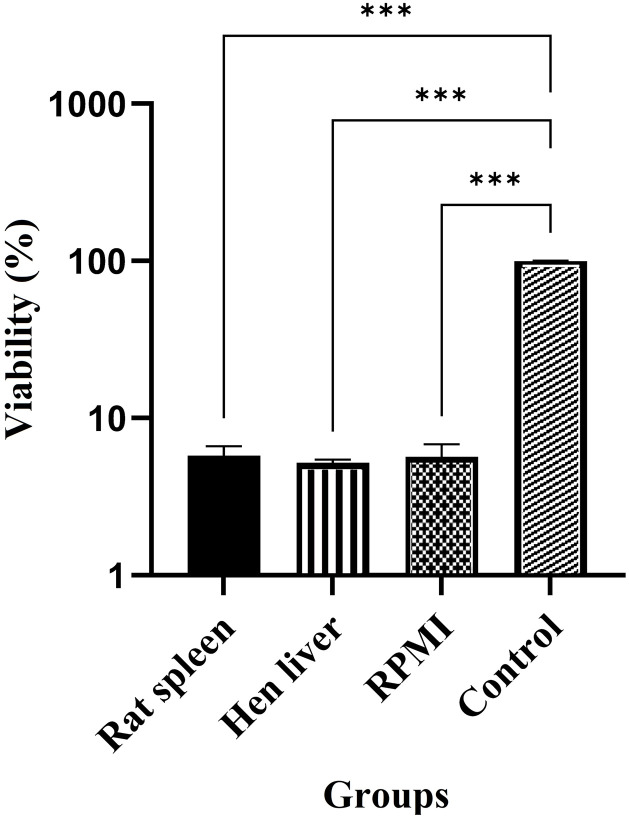
Comparison of the mean percentage of parasite survival across different study groups to identify the appropriate larval-parasite interaction environment. A one-way analysis of variance (ANOVA) test, corrected using Brown-Forsythe, was conducted at a 95% confidence level. The analysis compared three interactive groups (larvae/parasite/liver, larva/parasite/spleen, larva/parasite/RPMI) to the control group (parasite only).

To determine the optimal interaction environment, we co-incubated larvae and parasites for four hours in media containing the liver, spleen, and RPMI. The mean percentage of parasite survival in the treatment groups was significantly lower than that in the control group (*p* < 0.001). However, no significant differences were observed in pairwise comparisons between the treatment groups (*p* = 0.90; Fig 4). As no notable differences were detected among the interaction groups, subsequent experiments were conducted by exposing larvae to parasites in RPMI medium for four hours.

### Larval-parasite pre-oral interactions

**Fig 5:**
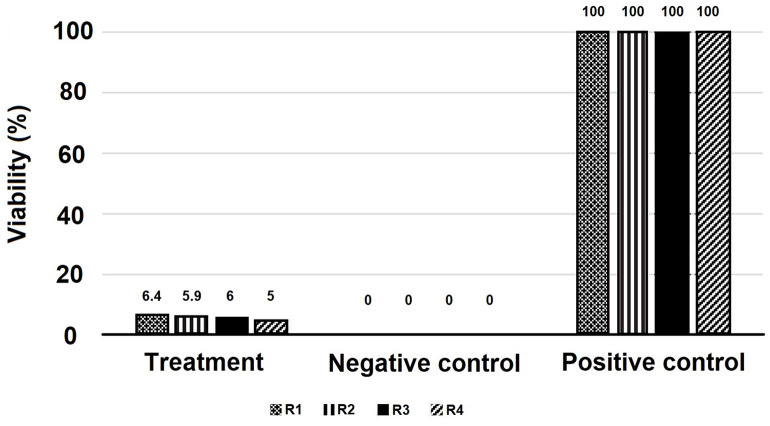
Comparison of the mean percentage of viable parasites between the intervention group (*L. sericata* and *L. major* in RPMI 1640 supplemented with 10% serum) and the control group (*L. major* in RPMI 1640 supplemented with 10% serum) outside the larval body, following a four-hour interaction. The viability of parasites was assessed using the trypan blue exclusion method.

**Fig 6:**
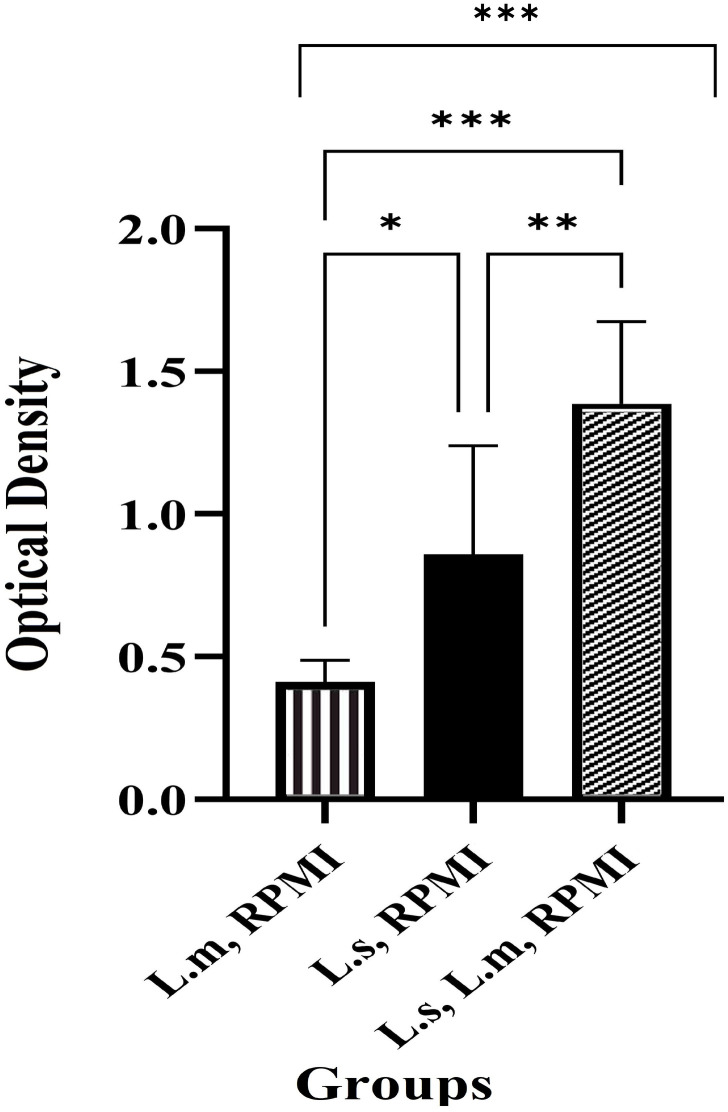
Comparison of the metabolically active cells among the treatment group (*L. sericata* and *L. major* in RPMI 1640 medium supplemented with 10% serum), negative control group (*L. sericata* in RPMI 1640 medium with 10% serum), and positive control group (*L. major* in RPMI 1640 medium with 10% serum) outside the larval body, following a four-hour interaction. The cell activities were assessed using the MTT assay.

The survival and activity of *L. major* outside the larval body were influenced by larval ES products or microbiota (Fig 2). A one-sample t-test revealed a significant decrease in the percentage of viable parasites in the treatment group compared to the positive control group (*L. major* alone) after four hours of exposure to the RPMI culture medium (*p* < 0.001; Fig 5; Fig 2A-C). Comparing the number of active cells among the intervention group, positive control (parasite only), and negative control (larvae only) using the MTT assay demonstrated significant differences between all three groups (*p* < 0.001; Fig 6). Unexpectedly, the number of active cells in both the intervention group and the negative control (larvae only) was higher than in the positive control (parasite only) group.

### Larval-parasite oral interactions

**Fig 7:**
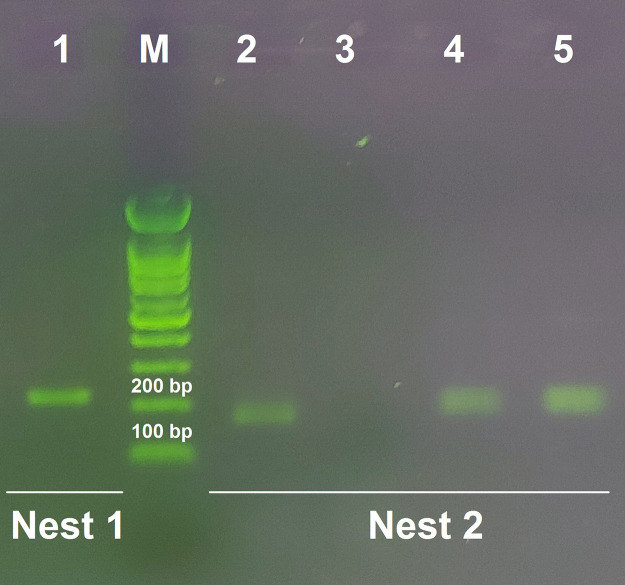
Species-specific nested PCR of *Leishmania major*, using the *ITS2* and *5.8S* gene. Lanes: 1, positive control (~213 bp); M, 100-bp ladder (Fermentas, USA); 2, positive control (~166 bp); 3, negative control (larval body); 4 and 5 (larvae exposed to *L. major*).

Microscopic examination of the digestive tracts of larvae exposed to parasites for four hours did not detect the presence of any live or dead parasites (Fig 2D-2F). To validate the microscopic findings and confirm the larvae’s ingestion of parasites by the larvae, nested PCR was conducted on 24 dissected larval digestive tracts, represented by eight triplicate pooled specimens. All specimens tested positive for parasite DNA in the second round of nested PCR (Fig 7; S1 Fig).

## Discussion

The present study investigated the interaction between *L. sericata* larvae and *L. major* parasites under intra-oral and extra-oral conditions to assess the potential of larval therapy as a biological tool for controlling *L. major* infections. The findings revealed that the regurgitated and/or defecated products of *L. sericata* larvae significantly influenced the survival and morphology of *L. major* parasites. Specifically, parasites exposed to larval ES products or microbiota exhibited morphological deformities within one hour post-exposure and underwent complete lysis six hours post-exposure. The survival percentage of parasites in the intervention groups decreased markedly over time compared to the control group, with no statistically significant differences observed among hen liver, rat spleen, and RPMI media. Furthermore, microscopic and molecular analyses confirmed that the larvae ingested *L. major* parasites and subsequently digested them within their digestive tracts. These results suggest that *L. sericata* larvae possess mechanisms that effectively neutralize *L. major* parasites, highlighting their potential as a promising therapeutic approach for managing leishmaniasis.

The observed deformations and lysis of *L. major* parasites are presumably mediated by the bioactive compounds present in the larval ES products or the regurgitated/defecated microbiota. Previous studies have demonstrated that larval ES contains antimicrobial peptides, proteolytic enzymes, and other bioactive molecules capable of disrupting microbial cell membranes and metabolic processes [32–34]. Similarly, the larval microbiota may contribute to parasite lysis by producing bacteriocins or other microbicidal substances. Consistent with this finding, the microbiota associated with the natural host of *Leishmania* parasites, the sandflies, influence the growth and survival of microorganisms within the vector [12,35]. For instance, specific bacterial genera, such as *Lysinibacillus*, *Serratia*, and *Pseudocitrobacter*, have been shown to significantly reduce the growth of *Leishmania* promastigotes in co-culture experiments [17].

In this study, we employed the trypan blue exclusion and MTT assays to assess the cell viability and metabolic activity of *L. major* parasites exposed to *L. sericata* larvae. Both assays are valuable tools in biomedical research; the trypan blue assay is simpler and faster, while the MTT assay provides more quantitative data. The MTT method can differentiate between healthy cells and those that appear viable but have lost their functionality. In contrast, the trypan blue method may fail to detect healthy cells in certain cases [36]. Interestingly, the MTT assay revealed higher viable cell counts in the intervention and negative control (larvae-only) groups, as compared to the positive control (parasite-only). Microscopic examination and culture on blood agar and LB broth media indicated that this discrepancy was likely due to larval microbiota being regurgitated or defecated into the environment (Fig 2D; bacteriological results not shown), which may have contributed to the increased metabolic activity observed in the MTT assay. These findings highlight the dual role of larval microbiota; while they assist in parasite elimination, their presence is required to be carefully considered when interpreting viability assays.

The four-hour exposure period was selected based on the observation that parasites became inactivated after this duration, ensuring sufficient time for larval-parasite interactions to occur. Furthermore, the absence of significant differences in parasite survival across the liver, spleen, and RPMI media suggests that the larval ES products or microbiota are robust enough to function effectively in various environments. This adaptability is particularly beneficial for potential clinical applications, as it indicates that larval therapy could be effective regardless of the tissue microenvironment.

Microscopic examination of larval digestive tracts revealed no live or dead *L. major* parasites, indicating complete digestion within the larval gut. The PCR results further confirmed the ingestion of parasites, as all tested samples were positive for *L. major* DNA. These findings align with previous studies demonstrating the ability of medicinal maggots to digest various pathogens, including bacteria and fungi [34]. The absence of intact parasites in the larval gut underscores the efficiency of larval digestive processes and supports the potential use of *L. sericata* larvae as a biological tool for eliminating *Leishmania* parasites. Our results are consistent with prior research on the antimicrobial properties of *L. sericata* larvae. In this context, studies have shown that larval ES products can inhibit the growth of bacterial pathogens such as *Staphylococcus aureus* and *Pseudomonas aeruginosa* [37]. However, this study expands these findings to include protozoan parasites, specifically *L. major*, highlighting the broad-spectrum efficacy of larval therapy. The rapid deformation and lysis of parasites observed in our study suggest that larval ES products or associated microbiota may target conserved cellular structures or pathways shared by diverse pathogens.

While the findings of this study provide compelling evidence for the anti-leishmanial effects of *L. sericata* larvae, several inherent limitations must be acknowledged. For instance, the exact composition and mechanistic insights of the larval ES products or microbiota remain unclear and warrant further investigation. Additionally, the present study was conducted under controlled laboratory conditions, and the efficacy of larval therapy in animal models [22] or human patients needs to be determined. Finally, given the complex interactions between *Leishmania* species and components of the immune system [38], potential immune responses induced by the maggots and their derivatives in the host organism should be examined to ensure safety and compatibility. Future studies should also consider investigating the synergistic effects of larval therapy combined with common anti-leishmanial drugs.

The results of this study, consistent with previous research, demonstrate that MDT can effectively reduce the microbial load in wounds by directly targeting *L. major* and secondary infectious bacteria. Additionally, MDT accelerates wound healing by mitigating the inflammatory phase induced by infectious agents. MDT has been standardized as a medical device and is recognized as a complementary method for treating chronic wounds in several countries, including the United States [39]. However, to date, no standardized protocol has been established for larval therapy targeting leishmaniasis. Development of a preliminary protocol for leishmaniasis-specific larval therapy requires meticulous consideration of various factors, such as patient selection criteria, application techniques, and post-therapy follow-up procedures. Table 1 outlines the essential requirements for creating such a protocol.

**Table 1:** A preliminary guideline for implementing maggot debridement therapy in patients with zoonotic cutaneous leishmaniasis, outlining patient selection criteria, larval preparation, application methods, monitoring procedures, and integration with conventional treatments, while specifying variations based on lesion type, size, depth, and patient health profile.

Steps	Requirements					
	1.1. Informed consent		MDT procedure			
			Free larvae	Biobag	Precautions	Contraindications
1. Patient inclusion/exclusion criteria and pre-treatment assessment	1.2. Health profile	1.2.1. Demographic factors (e.g., age, gender, ethnicity, education, and socio-economic status)	---	---	---	Up to 2 years After 85 years
		1.2.2. Vital signs (e.g., heart rate, blood pressure, temperature)	---	---	---	High temperature & ESR
		1.2.3. Immune system homeostasis	---	---	Atopic conditions (dermatitis, asthma, keratoconjunctivitis)	Autoimmune diseases, Insect bite
		1.2.4. Comorbidities	---	---	---	Cancer, Cardiovascular diseases, bleeding disorders, HIV/AIDS
		1.2.5. Mental health	---	---	---	Delusional parasitosis and entomophobia
	1.3. Lesion characteristics	1.3.1. Confirmed diagnosis (microscopy, PCR, or serological tests)	ZCL	ZCL	ACL	MCL, PKDL and VL
		1.3.2. Size (cm^2^)	>1	>4	>10	Closed wounds
		1.3.3. Depth (cm)	<5	<5	---	>5 Fistulae and canal that can’t prob at the end
		1.3.4. Stage (With or without necrosis/ infection)	Ulcer, crusts	Infiltration, Epithelialization, Desquamation, Erythema, Scaring	Papule-nodular and dry necrotic tissue	Papule
		1.3.5. Location	Trunk, shoulders, hands and legs	Face & toes	Neck and near the abdominopelvic cavity	Near to body cavities (eye, mouth and nose) and the main vessels
		1.3.6. Chemotherapy	Cases refractory to antileishmanials	Cases refractory to antileishmanials	Chemotherapy	---
	1.4. Lifestyle factors	1.4.1. Habit	Proper nutrition	Proper nutrition	Pressure ulcers, stress, poor hygiene	Cigarette smoking, Alcohol consumption
		1.4.2. Physical activity	---	---	---	Heavy sports
		BMI	---	<18.5 kg/m^2^	Obese class 1–3	---
2. Preparation of larvae	2.1. Species selection		2.1.1. Use sterile *L. sericata* larvae reared under controlled conditions to ensure safety and efficacy.			
			2.1.2. Verify the larvae’s sterility through microbiological testing before application.			
	2.2. Larval size and quantity		2.2.1. Select larvae in the second instar stage (~2 mm in size) for optimal activity.			
			2.2.2. Calculate the number of larvae based on lesion size (typically 1–5 larvae per cm² of wound area).			
	2.3. Sterilization process		2.3.1. Sterilize surface of eggs using proper disinfectants.			
			2.3.2. Grow up larvae in a sterile environment with a sterile (blood agar) diet.			
			2.3.3. Sterilize surface and gut of larvae again, post-hatching.			
			2.3.4. Check the quality control rigorously to confirm sterility and viability.			
	2.4. Storage and transport		2.4.1. Store larvae at 4°C until use to slow their metabolism without harming them.			
			2.4.2. Transport larvae in sterile containers with a microbiological filter and moist substrate (e.g., hydrogel, blood agar or gauze) to prevent desiccation			
3. Application of larvae	3.1. Wound Preparation		3.1.1. Clean the lesion with saline solution to remove debris and crusts.			
			3.1.2. Avoid using antiseptics or antibiotics that may harm the larvae.			
	3.2. Application method		3.2.1. Place larvae directly or in the biobag onto the lesion surface based on above considerations.			
			3.2.2. Cover the area with a breathable, occlusive dressing (e.g., mesh or netting) to contain the larvae while allowing airflow.			
			3.2.3. Secure the area dressing with adhesive bandages or wraps to prevent larvae from escaping.			
	3.3. Duration of larval therapy (24/48 hours apply/rest intervals)		3.3.1. Leave larvae in place for 12–24 hours, depending on lesion severity and response.			
			3.3.2. Replace larvae every 48 hours if multiple cycles are needed.			
4. Monitoring and evaluation	4.1. Clinical parameters		4.1.1. Measure lesion size, depth, and appearance daily or after each larval cycle.			
			4.1.2. Assess for signs of improvement, such as reduced necrosis, granulation tissue formation, and decreased inflammation			
	4.2. Parasite load		4.2.1. Perform microscopic examination or PCR assay of lesion samples before and after treatment to evaluate parasite clearance			
	4.3. Patient feedback		4.2.2. Monitor for adverse effects (e.g., itching, discomfort) and address any concerns promptly			
	4.4. Outcome measures		4.4.1. Primary outcome: Complete wound healing and resolution of infection.			
			4.4.2. Secondary outcomes: Reduction in lesion size, parasite load, and time to healing			
5. Integration with conventional therapies	5.1. Adjunctive use		5.1.1. Combine MDT with systemic antileishmanial drugs (e.g., sodium stibogluconate, miltefosine) for synergistic effects.			
			5.1.2. Administer local treatments (e.g., topical antimicrobials) if secondary bacterial infections are present			
	5.2. Sequential therapy		5.2.1. Use MDT as an initial debridement step to prepare the wound bed before starting drug therapy.			
			5.2.2. Alternatively, apply MDT after completing conventional treatment to promote final wound closure.			

**Abbreviations:** Cl: cutaneous leishmaniasis, MCL: Mucocutaneous leishmaniasis, MDT: maggot debridement therapy, PKDL: Post kala-azar dermal leishmaniasis. ESR: Erythrocytes sedimentation rate, BMI: Body mass index.

## Conclusion

This study demonstrates the potent anti-leishmanial activity of *L. sericata* larvae, mediated by their ES products or associated microbiota. The rapid deformation, inactivation, and lysis of *L. major* parasites and the complete digestion of ingested parasites within the larval gut underscore the potential of larval therapy as an innovative approach to treating leishmaniasis. Our in vitro and preliminary findings support the development of larval therapy protocols for clinical application in dermatology, offering a novel strategy for managing chronic wounds associated with ZCL. Further in vivo studies or clinical trials are necessary to elucidate the underlying specific mechanisms and translate these findings into clinical applications.

## Supporting information

S1 Fig Original, uncropped and minimally adjusted image for Fig 7. Species specific nested PCR of *Leishmania major*, using the ITS2 and 5.8S gene. Lanes: 1, positive control (~213 bp); M, 100-bp ladder (Fermentas, USA); 2, positive control (~166 bp); 3, negative control (larval body); 4 and 5 (larvae exposed to *L. major*).
(PDF)
