# Supplementary material for: From pre-oral secretions to gut digestion: How do Lucilia sericata (Diptera: Calliphoridae) larvae handle Leishmania major?
Source: PLoS One. 2025 Oct 22;20(10):e0334553. doi: 10.1371/journal.pone.0334553 (PMC12543111; doi:10.1371/journal.pone.0334553)
Supplement: S1 Fig — Lanes: 1, positive control (~213 bp); M, 100-bp ladder (Fermentas, USA); 2, positive control (~166 bp); 3, negative control (larval body); 4 and 5 (larvae exposed to L. major). (PDF) [file pone.0334553.s001.pdf]

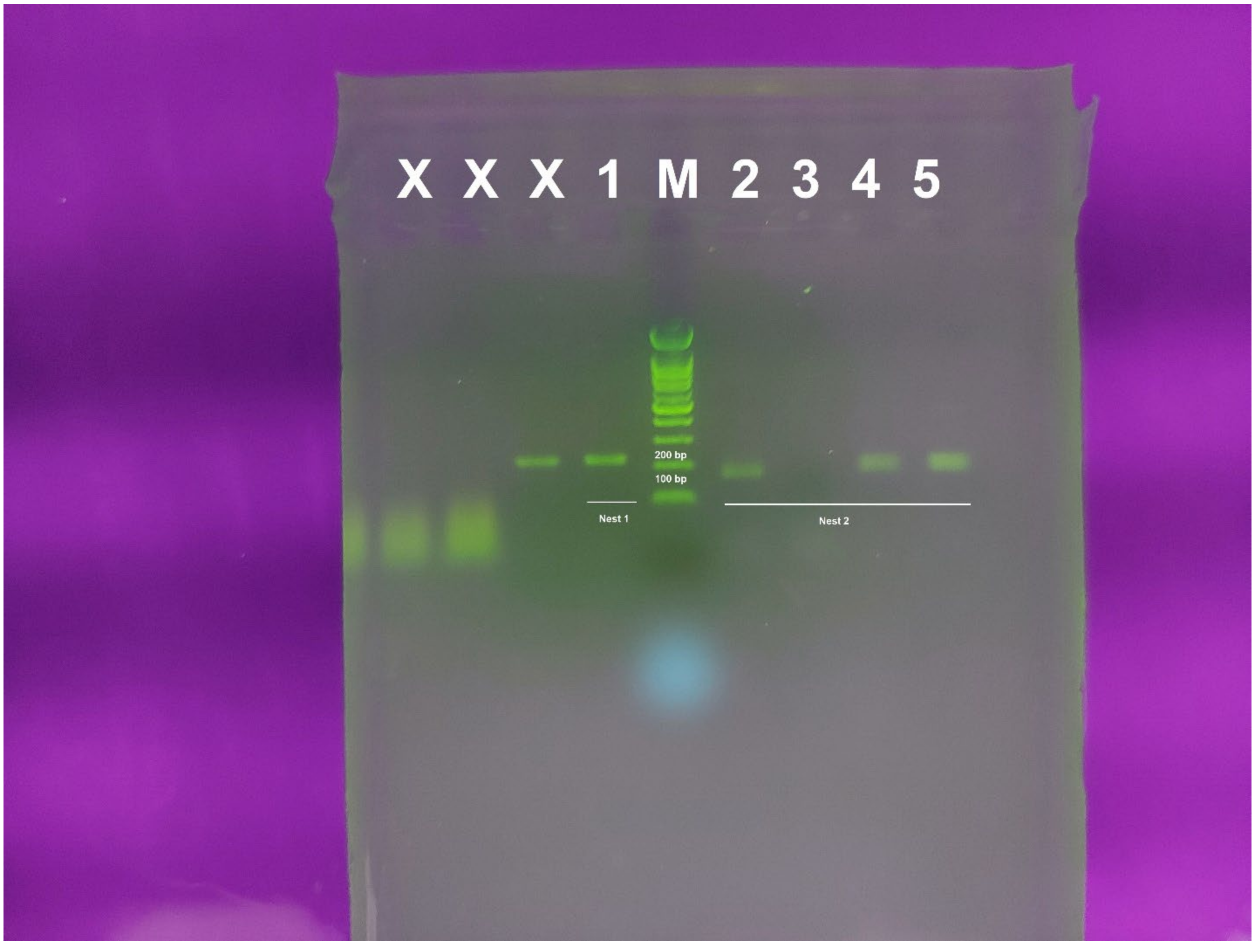

Supplementary Fig 1. Original, uncropped and minimally adjusted image for Fig 7: Species-specific nested PCR of *Leishmania major*, using the *ITS2* and *5.8S* gene. Lanes: 1, positive control (~213 bp); M, 100-bp ladder (Fermentas, USA); 2, positive control (~166 bp); 3, negative control (larval body); 4 and 5 (larvae exposed to *L. major*).
